# Associating liver partition and portal vein ligation for staged hepatectomy versus conventional two-stage hepatectomy: a systematic review and meta-analysis

**DOI:** 10.1186/s12957-017-1295-0

**Published:** 2017-12-19

**Authors:** Zheng Zhou, Mingxing Xu, Nan Lin, Chuzhi Pan, Boxuan Zhou, Yuesi Zhong, Ruiyun Xu

**Affiliations:** Department of Hepatobiliary Surgery, Third Affiliated Hospital of Sun Yat-sen University, No. 600 Tianhe Road, Guangzhou, Guangdong China

**Keywords:** ALPPS, Two-stage hepatectomy, Meta-analysis

## Abstract

**Background:**

It is generally accepted that an insufficient future liver remnant is a major limitation of large-scale hepatectomy for patients with primary hepatocellular carcinoma. Conventional two-stage hepatectomy (TSH) is commonly considered to accelerate future liver regeneration despite its low regeneration rate. Associating liver partition and portal vein ligation for staged hepatectomy (ALPPS), which is characterized by a rapid regeneration, has brought new opportunities.

**Methods:**

Relevant studies were identified by searching the selected databases up to September 2017. Then, a meta-analysis of regeneration efficiency, complication rate, R0 resection ratio, and short-term outcomes was performed.

**Results:**

Ten studies, comprising 719 patients, were included. The overall analysis showed that ALPPS was associated with a larger hyperplastic volume and a shorter time interval (*P* < 0.00001) than TSH. ALPPS also exhibited a higher completion rate for second-stage operations (odds ratio, OR 9.50; *P* < 0.0001) and a slightly higher rate of R0 resection (OR 1.90; *P* = 0.11). Interestingly, there was no significant difference in 90-day mortality between the two treatments (OR 1.44; *P* = 0.35).

**Conclusions:**

These results indicate that compared with TSH, ALPPS possesses a stronger regenerative ability and better facilitates second-stage operations. However, the safety, patient outcomes, and patient selection for ALPPS require further study.

**Electronic supplementary material:**

The online version of this article (10.1186/s12957-017-1295-0) contains supplementary material, which is available to authorized users.

## Background

Multiple and large liver cancers remain a major challenge in liver surgery, although hepatectomy has become the most effective treatment [1]. A very common reason is the limited future liver remnant (FLR), one of the determining factors leading to postoperative liver failure (PLF), which restricts the application of this method. Commonly, a volume of over 20% FLR must remain to avoid PLF in a normal liver [2]. Conventional two-stage hepatectomy (TSH), including portal vein embolization (PVE) or portal vein ligation (PVL) accompanied by subsequent hepatectomy, represents one solution to this dilemma [3]. Regrettably, the shortcomings of a long time interval and low regeneration efficiency seriously limit its application [4]. In 2012, a new surgical approach named “associating liver partition and portal vein ligation for staged hepatectomy (ALPPS)”, characterized by great liver regeneration efficiency, was proposed by Schnitzbauer et al. [5]. However, standard methods for patient selection and the long-term outcomes of this new approach remain controversial.

Several systematic reviews have examined the advantages and disadvantages of ALPPS, especially the ability to promote an increased FLR. However, this method continues to provoke heated debate because of its high mortality and unclear feasibility compared with other technologies, such as conventional TSH. Moreover, patient outcomes of ALPPS also remain uncertain [6–8]. In the current analysis, we systematically searched for published studies comparing ALPPS and TSH to evaluate the liver regeneration efficiency, safety, and complication rates of these two methods.

## Methods

The prospective agreement on study objectives, literature search methods, inclusion and exclusion criteria, outcome measurements, and statistical analysis methods were selected according to the Preferred Items for Systematic Reviews and Meta-Analysis (PRISMA) (Additional file 1: Appendix S1) and Meta-Analysis of Observational Studies in Epidemiology (MOOSE) guidelines (Additional file 2: Table S1).

There is currently no agreement on the definition of TSH. For the purpose of statistical analysis, the TSH group included patients who received PVE, PVL, or both.

### Search strategy and selection criteria

A search of the PubMed, Embase, and Cochrane Library databases was performed to identify all studies comparing ALPPS and TSH. The following terms were searched: “liver partition,” “liver transection,” “portal vein occlusion,” “PVO,” “portal vein embolization,” “PVE,” “portal vein ligation,” “PVL,” “associating liver partition and portal vein ligation for staged hepatectomy,”, “ALPPS,” “staged hepatectomy,” and “two-stage hepatectomy.” The publication time was from January 1, 2009, to September 30, 2017, and the language was restricted to English.

All comparative studies comparing ALPPS with TSH in primary or secondary liver tumor patients who received surgery for staged hepatectomy were included. These studies included at least one quantitative outcome of interest, such as regeneration efficiency, time interval of the two stages, completion rate for second-stage operations, tumor deterioration, insufficient regeneration, R0 resection ratio, liver failure, bile leak, 90-day mortality, and 1-year disease-free survival. The excluded studies were those with irrelevant topics, case reports, non-comparative studies, review articles, letters, incomplete multiple published reports, and conference abstracts.

### Data extraction and outcomes of interest

Two authors independently extracted and summarized the following data: patient characteristics, research designs, inclusion and exclusion criteria, and reported outcomes. Any disagreements were jointly resolved by the authors.

### Quality assessment and statistical analysis

The studies ultimately included were classified according to the “2011 Levels of Evidence for Common Harms (Treatment Harms)” (Centre for Evidence Based Medicine, Oxford, UK). These levels of evidence can be described as follows: systematic reviews of randomized trials, systematic reviews of nested case-control studies and n-of-1 trials (level 1); individual randomized trials or (exceptionally) observational studies with a dramatic effect (level 2); non-randomized controlled cohort/follow-up studies, provided there are sufficient numbers to rule out a common harm (level 3); case-series, case-control studies, or historically controlled studies (level 4); and mechanism-based reasoning (level 5) [9].

The weighted mean difference (WMD) and OR were used to compare continuous and dichotomous variables, respectively. The OR and 95% confidence interval (CI) were calculated for binary data. *P* values < 0.05 were considered to indicate statistical significance. When the mean and variance were not reported, they were calculated using the median and range through a formula reported by Hozo SP [10]. Heterogeneity between studies was assessed using the chi-squared test and *I* ^2^. A *P* value < 0.10 was used to indicate heterogeneity. Fixed-effect models were used for cumulative analyses when there was a lack of heterogeneity; otherwise, random-effect models were used.

The quality of all included studies was assessed using the Newcastle-Ottawa Scale (NOS) system, and patient selection, study comparability, and outcomes were evaluated. Scores ranging from 0 to 9 were calculated for each included study, and studies achieving a score of 6 or more were considered to be of good quality. Statistical analysis was performed using Review Manager Version 5.2 software (Cochrane Collaboration, Oxford, UK) (Additional file 3: Table S2).

## Results

**Fig. 1:**
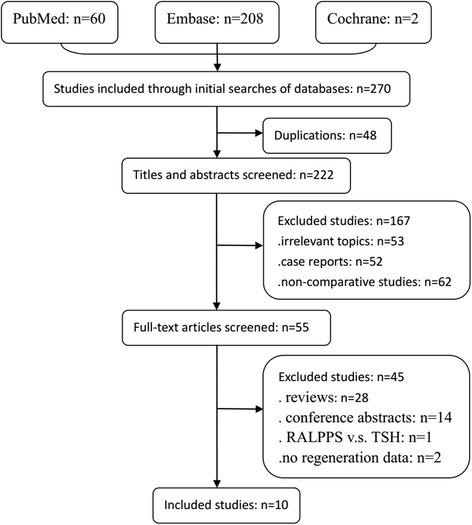
Flowchart for the selection of eligible studies

Figure 1 shows the results of the search strategy and all studies that were included and excluded. In total, 270 studies were identified from the databases. Forty-eight studies were excluded because of duplication, and 167 studies were excluded after the titles and abstracts had been reviewed. The full-text articles of 55 studies were screened in detail. Of these articles, 28 reviews were excluded, 14 conference abstracts were excluded, and 1 study comparing radiofrequency-ALPPS (RALPPS) and TSH was excluded. Another 2 studies were excluded due to the lack of regeneration data. Between the two reviewers, there was 95% agreement for study selection and 96% agreement for quality assessment of the trials.

### Characteristics and quality of the included studies

Ten studies were included according to the inclusion criteria [11–20]. Table 1 shows the clinical characteristics of these studies. Nine of the studies were retrospective articles, and only one randomized controlled trial (RCT) was identified. The references of these included studies indicated that no other studies existed with which to assess the topic further. In total, 201 patients in the ALPPS group and 518 patients in the TSH group were analyzed in this meta-analysis. Six studies compared ALPPS with PVE/PVL, and 4 studies compared ALPPS with PVE only. Two studies comprised a possible overlapping population, but they were both included because they investigated different outcomes [15, 20]. Complete short-term follow-up data were available in two studies. The preoperative chemotherapy rate was 48–100% in the ALPPS group and 53–87% in the TSH group.

**Table 1:** Clinical and pathological characteristics of the included studies

Author	Year	No. of patients	Preoperative chemotherapy	Tumor size (mm)	TNM stage	Comparable variables	Level	Study design	Quality score
Knoefel et al. [14]	2012	ALPPS(7) PVE(15)	5 NI	NI	NI	1, 3, 7	4	RCS	6
Shindoh et al. [19]	2013	ALPPS(25) PVE(144)	12 94	NI	NI	1, 2, 5, 7	3	RCS	7
Croome et al. [13]	2014	ALPPS(15) PVE(53)	14 40	NI	NI	1, 2, 5, 7, 9	3	RCS	8
Schadde et al. [18]	2014	ALPPS(48) PVE/PVL(83)	28 44	NI	NI	1, 2, 4, 5, 7, 8, 9	3	RCS	9
Ratti et al. [16]	2015	ALPPS(12) PVE/PVL(36)	9 30	NI	NI	1, 2, 5, 6, 7, 8, 9	3	RCS	9
Matsuo et al. [15]	2015	ALPPS(8) PVE(14)	8 12	21 35.5	NI	1, 2, 3, 5, 7	4	RCS	6
Tanaka et al. [20]	2015	ALPPS(11) PVE/PVL(54)	11 47	22 40.5	NI	1, 2, 7	4	RCS	7
Adam et al. [11]	2016	ALPPS(17) PVE/PVL(41)	17 41	40 50	NI	1, 2, 5, 6, 7, 8	4	RCS	7
Chia et al. [12]	2017	ALPPS(10) PVE/PVL(29)	3 16	81 66	NI	1, 2, 4, 5, 7	4	RCS	7
Sandström et al. [17]	2017	ALPPS(48) PVE/PVL(49)	NI NI	54 49	NI	1, 2, 3, 5, 7, 8, 9	1	RCT	8

1: age; 2: preoperative chemotherapy; 3: preoperative liver function; 4: pathology state before operation; 5: pathology type of tumor; 6: pathology stage; 7: sex; 8: ASA score; 9: BMI

*NI* no information, *RCS* retrospective cohort study, *RCT* randomized controlled trial

### FLR regeneration

**Fig. 2:**
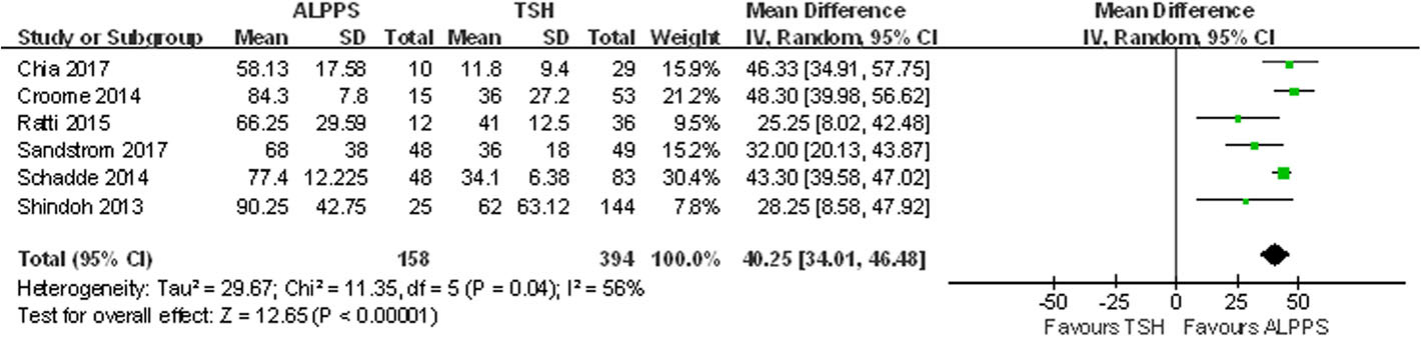
Forest plot and meta-analysis of the absolute value of future liver remnant regeneration

Figure 2 summarizes the absolute value of FLR regeneration in the 6 included studies. The results of the random-effect model indicated that ALPPS led to greater regeneration than TSH did (WMD 40.25; 95% CI, 34.01~46.48; *P* < 0.00001).

### Time interval of the two stages

**Fig. 3:**
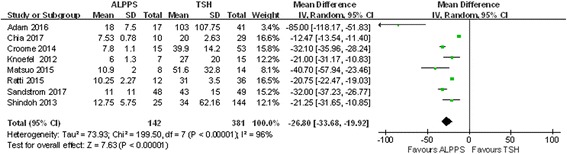
Forest plot and meta-analysis of the time interval between the two stages

Eight studies were included in this section, and the results are shown in Fig. 3. The duration before the second-stage operation was shorter for ALPPS than for TSH (WMD − 26.80; 95% CI,− 33.68~− 19.92; *P* < 0.00001); however, significant heterogeneity was observed (chi-square = 199.50; df = 7; *P* < 0.00001; *I* ^2^ = 96%).

### Completion rate of second-stage operations

**Fig. 4:**
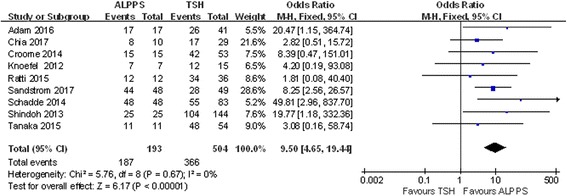
Forest plot and meta-analysis of the second surgery completion rate

Nine studies included information on the completion rate for second-stage operations. There was no apparent heterogeneity in these studies (chi-square = 5.76; df = 8; *P* = 0.67; *I* ^2^ = 0%). The completion rate of second-stage operations was 96.89% in ALPPS and 72.62% in TSH when analyzed with a fixed model (OR 9.50; 95% CI, 4.65~19.44; *P* < 0.00001) (Fig. 4), and this difference was statistically significant.

**Fig. 5:**
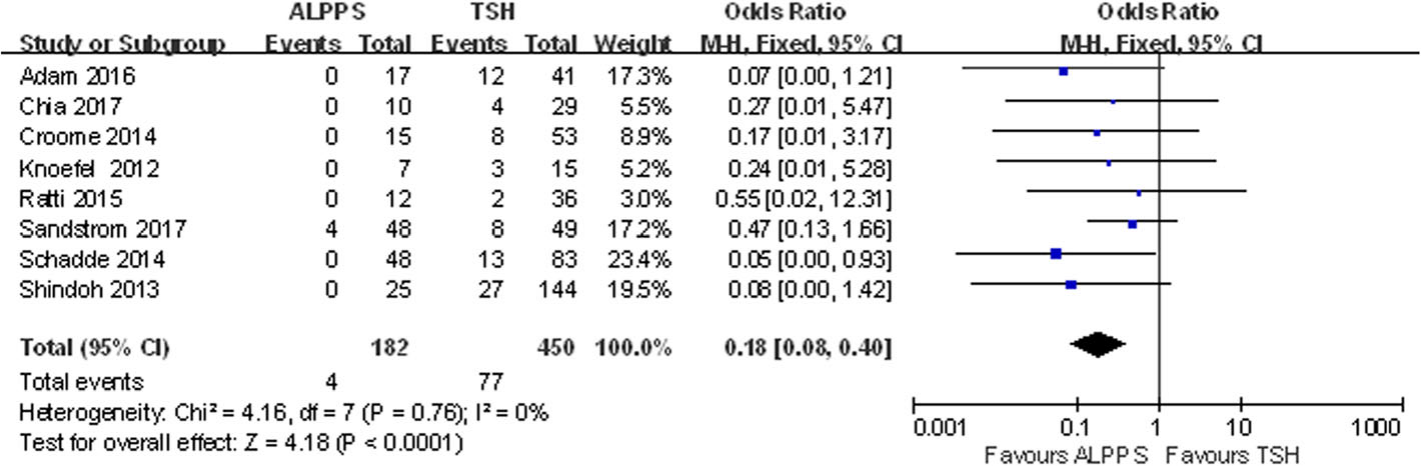
Forest plot and meta-analysis of tumor progression in patients who failed to receive a second operation

**Fig. 6:**
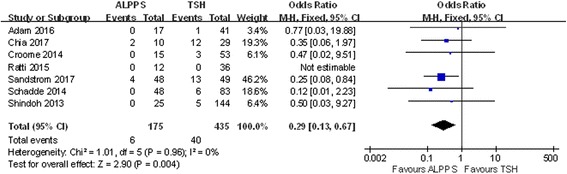
Forest plot analysis of the insufficient regeneration rate in patients who failed to receive a second operation

The reasons for failure to complete the staged operations included tumor progression and insufficient FLR. Eight studies were included, and only 2% of patients experienced tumor progression before the second operation in ALPPS, lower than the 17.1% in TSH. (OR 0.18; 95% CI, 0.08~0.40; *P* < 0.0001) (Fig. 5). The difference in the insufficient regeneration rate between ALPPS and TSH was also significant, with TSH showing a higher rate than ALPPS (OR 0.29; 95% CI, 0.13~0.67; *P* = 0.0004) (Fig. 6).

### Postoperative complications

**Fig. 7:**
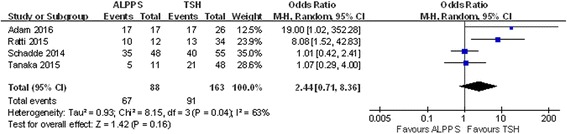
Forest plot analysis of postoperative complications in patients after stage 2 operation

**Fig. 8:**
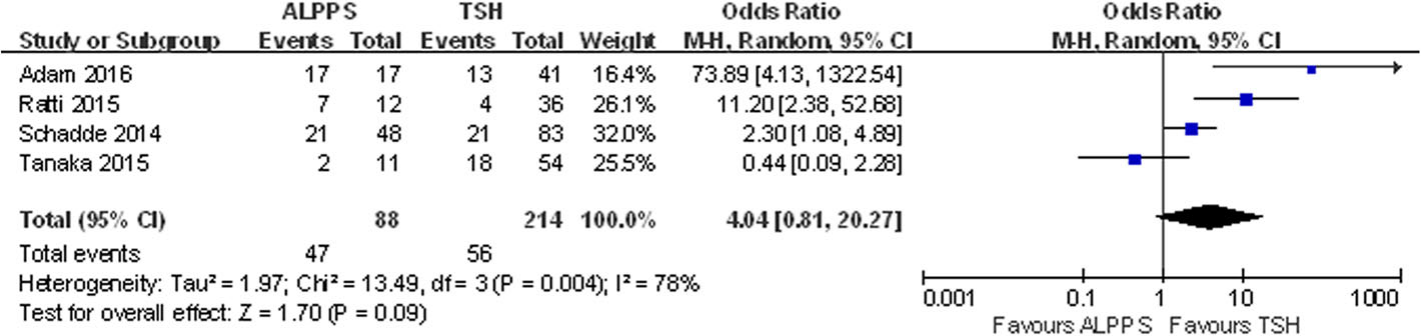
Forest plot analysis of postoperative complications in patients after stage 1 operation

**Fig. 9:**
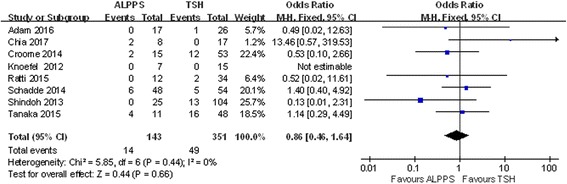
Forest plot and meta-analysis of the postoperative liver failure rate in patients after stage 2 operation

**Fig. 10:**
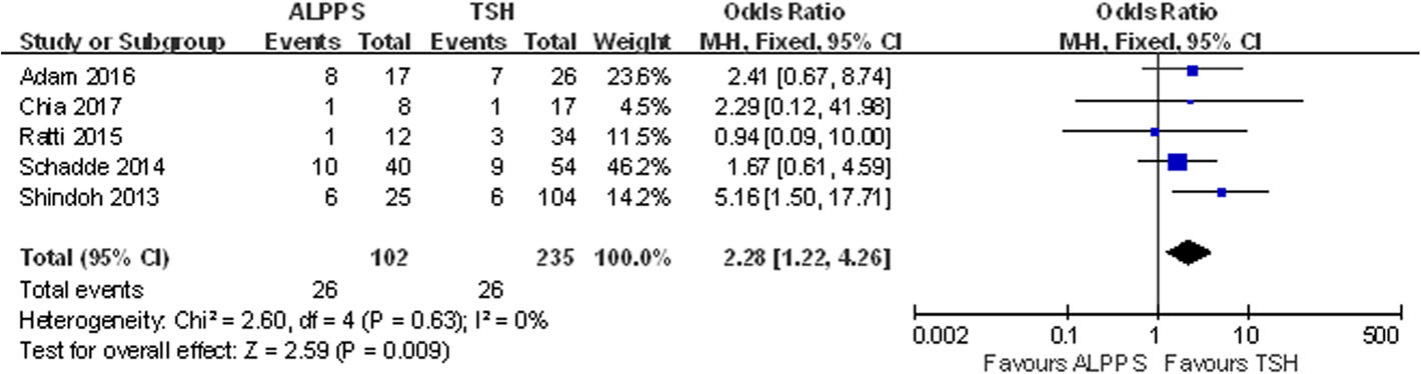
Forest plot and meta-analysis of the bile leak rate in patients after stage 2 operation

**Fig. 11:**
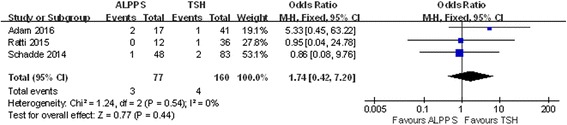
Forest plot and meta-analysis of the bile leak rate in patients after stage 1 operation

The incidence of postoperative complications was higher in the ALPPS group after two stages, yet no significant differences were observed (Figs. 7 and 8). Eight studies included PLF data. In the ALPPS group, 9.80% of patients experienced liver failure, and this percentage was lower than the 13.96% observed in the TSH group, although this difference was not significant (OR 0.86; 95% CI, 0.46~1.64; *P* = 0.66) (Fig. 9). Another 5 studies mentioned postoperative bile leaks. In these studies, we observed a higher bile leak rate after the stage 2 operation in the ALPPS group (OR 2.28; 95% CI, 1.21~4.26; *P* = 0.009) (Fig. 10), and a similar conclusion could be drawn after stage 1, although the difference was not significant (OR 1.74; 95% CI, 0.42~7.20; *P* = 0.44) (Fig. 11).

### R0 resection and 1-year disease-free survival

**Fig. 12:**
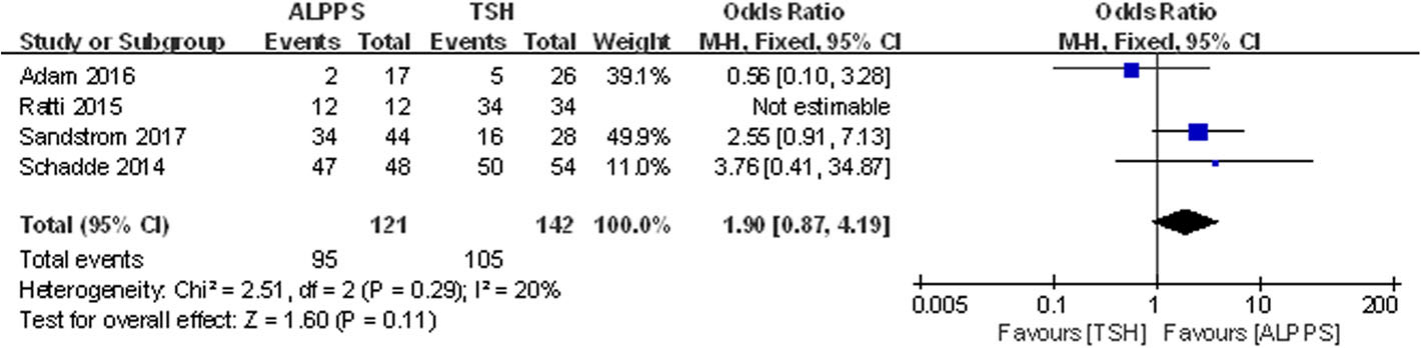
Forest plot and meta-analysis of the R0 resection ratio

**Fig. 13:**
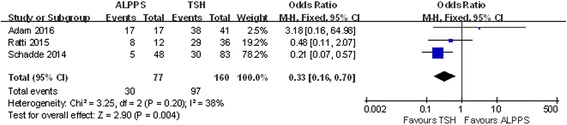
Forest plot and meta-analysis of 1-year disease-free survival

Four studies included data on R0 resection and 1-year disease-free survival, and no heterogeneity was observed in these studies (chi-square = 2.51; df = 2; *P* = 0.29; *I* ^2^ = 20%). The results showed that in the ALPPS group, 78.51% of patients obtained R0 resection, which was slightly higher than the 73.94% in the TSH group, although the difference was not statistically significant (OR 1.90; 95% CI, 0.87~4.19; *P* = 0.11) (Fig. 12). Additionally, ALPPS showed a lower 1-year disease-free survival rate than TSH did (OR 0.33; 95% CI, 0.16~0.70; *P* = 0.004) (Fig. 13).

### Short-term outcomes

**Fig. 14:**
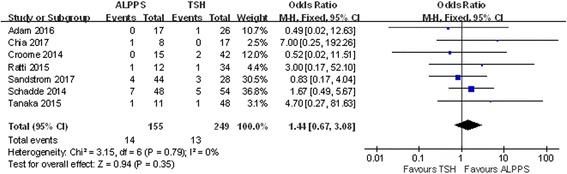
Forest plot and meta-analysis of 90-day mortality after stage 2 operation

There were 7 studies related to this topic, and no heterogeneity between studies was identified (chi-square = 3.15; df = 6; *P* = 0.79; *I* ^2^ = 0%). Although the 90-day mortality was higher for patients receiving ALPPS (OR 1.44; 95% CI, 0.67~3.08), this difference was not statistically significant (*P* = 0.35) (Fig. 14).

## Discussion

This systematic review and meta-analysis included 719 patients and aimed to compare the regeneration efficiency, safety, and complication rates of ALPPS and TSH. The absolute value of FLR regeneration in ALPPS was significantly higher than that in TSH, and the interval of the two stages in ALPPS was clearly shorter than that in TSH. In addition, ALPPS was associated with a higher completion rate, a lower probability of tumor progression during the stage interval, and a lower insufficient regeneration rate; these findings are similar to those of previous studies [21–23]. However, complications, especially bile fistulas, were much more frequent in ALPPS, likely because of the liver splitting required in this procedure. Although ALPPS was associated with a lower rate of 1-year disease-free survival, there was no significant difference in the 90-day mortality rate between these two methods.

Given the safety of operation and possibility of PLF, a surgeon’s decision regarding a secondary ALPPS surgery is often affected by various factors. Two common factors are residual liver volume and function. In general, once the volume of liver regeneration reaches 20% of the normal liver and 30% of the cirrhotic liver, secondary surgery can be safely performed [13–16, 18–20]. The rate of FLR regeneration differs during the regeneration progress. For instance, Correa reported that for 10 patients who did not accept resection but received PVE, the FLR regeneration rate was different at various periods within the 1-year follow-up, and the liver continued to grow even 1 year after PVE [24]. However, the FLR regeneration volume alone still cannot reflect effective liver regeneration since patients usually have different regeneration rates after the operation. In our study, the absolute value of FLR regeneration was higher in ALPPS than in TSH, similar to the findings of previous studies. However, there was no uniform calculation of kinetic growth rate (KGR), and we did not perform a unified analysis of these included studies to better assess liver regeneration.

Furthermore, the pathological state of the liver also influences the potential for regeneration. Commonly, insufficient regeneration is accompanied by chronic liver disease. Chia et al. reported that patients with hepatitis B virus (HBV)-related cirrhosis require a longer time for regeneration before stage 2 [25]. Furthermore, neoadjuvant chemotherapy and chemotherapy have been reported to inhibit liver regeneration to some extent.

What is the best way to evaluate liver regeneration? In 2013, Shindoh et al. proposed the concept of KGR to try to resolve this issue [26]. However, KGR may reflect only the increased volume, not the increased function of the liver. Moreover, there is no uniform calculation for KGR, and therefore, this index remains controversial. Additionally, several studies have shown that many cytokines expressed during the liver regeneration process following ALPPS are associated with regeneration [27–29]. Portal hemodynamics and immature hepatocytes during the process were both considered influencing factors [15, 21, 30, 31]. Given all these factors, the most effective method to evaluate the function of the FLR remains unclear and further work is necessary to address the timing and selection of optimal candidates for staged hepatectomy.

Additionally, the interval time in TSH was longer, and tumor progression was more common [32]. It is possible that the long time interval of TSH caused a higher risk of tumor progression. Only 4 patients were diagnosed with tumor progression in the ALPPS group in this study. Moreover, Fukami reported that Ki-67, a marker of cell proliferation, was increased to 80% in tumor cells after the second stage of ALPPS, and this increase was higher than that identified in the first stage in patients with colon cancers and synchronous multiple liver metastases [33]. However, there is no consensus regarding whether the rapid regeneration response in ALPPS can aggravate the progression of the primary tumor. In addition, some researchers have reported that the incidence of postoperative tumor recurrence ranges from 14 to 87%, which is higher than that of TSH. Our study also indicated a similar conclusion. Furthermore, a higher R0 ratio could be seen in the ALPPS group; this result may be related to the entire splitting of stage 1. Additionally, there is no benefit to extending the interval time before stage 2, as a decreased KGR may occur at the seventh day after stage 1 [34]. At the same time, considering that performing the stage 2 operation without sufficient preoperative assessment of the liver regeneration may easily lead to liver failure, it is necessary to establish an evaluation standard for assessing liver function after stage 1.

The mortality and complication rate are believed to be higher following ALPPS than following TSH. Our results also showed this trend although there was no significant difference between the two groups. Commonly increased portal blood, the most distinctive characteristic differentiating ALPPS from TSH, would lead to portal hyperperfusion which can conversely cause liver failure. Allard et al.’s research has shown a positive correlation between the portal vein pressure (PVP) and 90-day mortality. A multivariate analysis also indicated that PVP after hepatectomy was an independent predictor for PLF [35]. Thus, the hemodynamic change in the portal vein after hepatectomy is closely related to patient prognosis. The increased portal vein flow in ALPPS can block the circulation from two parts of the liver and result in centralized hepatic blood flow, which ultimately leads to portal hyperperfusion of the FLR [36]. However, with the hepatic artery buffering effect, the arterial flow in the non-ligated portion decreases to partly adapt to the increased portal vein flow [37]. Vicente et al. investigated the pathological specimens of small-size syndrome patients and found that sinusoidal dilatation of the tissue was directly related to liver failure [38].

There are many limitations of this meta-analysis. First, most of the included studies presented a low level of evidence, ranging from level 3 to level 4. Second, there was significant heterogeneity in some of the results because of the differences across studies in institutions, numbers of patients, types of primary tumors, and other factors. Third, there was obvious publication bias among some of the included studies.

## Conclusions

In conclusion, this meta-analysis found that ALPPS was associated with a higher regeneration efficiency and a higher operation completion rate than TSH. Patients receiving TSH were more likely to experience tumor progression during the time interval. ALPPS also provided a higher rate of R0 resection, although the rate of complications after stage 2 was higher than that of TSH. However, the long-term outcome of ALPPS remains unclear, and additional studies are needed to address this issue.

## Additional files

Additional file 1: Appendix S1. PRISMA 2009 Checklist. (DOC 64 kb)

Additional file 2: Table S1. MOOSE Checklist. (DOCX 21 kb)

Additional file 3: Table S2. Assessment of quality. (DOCX 15 kb)

## Abbreviations

ALPPS: Associating liver partition and portal vein ligation for staged hepatectomy
CI: Confidence interval
FLR: Future liver remnant
KGR: Kinetic growth rate
NOS: Newcastle-Ottawa Scale
OR: Odds ratio
PLF: Postoperative liver failure
PVE: Portal vein embolization
PVL: Portal vein ligation
PVO: Portal vein occlusion
PVP: Portal vein pressure
RALPPS: Radiofrequency-ALPPS
TSH: Two-stage hepatectomy
WMD: Weighted mean difference
