# Supplementary material for: Associating liver partition and portal vein ligation for staged hepatectomy versus conventional two-stage hepatectomy: a systematic review and meta-analysis
Source: World J Surg Oncol. 2017 Dec 19;15:227. doi: 10.1186/s12957-017-1295-0 (PMC5738171; doi:10.1186/s12957-017-1295-0)
Supplement: Supplementary file 2 — MOOSE Checklist. (DOCX 21 kb) [file 12957_2017_1295_MOESM2_ESM.docx]

**Table S1 MOOSE Checklist**

| **Criteria** | | **Brief description of how the criteria were handled in the meta-analysis** |
| --- | --- | --- |
| **Reporting of background should include** | |  |
| √ | Problem definition | The association between ALPPS and TSH still remains unclear, especially for selecting the better strategy on promoting liver regeneration in liver cancer. |
| √ | Hypothesis statement | ALPPS has a stronger regenerative ability and can easily implement the second stage operation than TSH. |
| √ | Description of study outcomes | regeneration efficiency, complication rate, R0 resection ratio, and short-term outcome. |
| √ | Type of exposure or intervention used | Different methods (ALPPS and TSH). |
| √ | Type of study designs used | Cohort studies and case-control studies. |
| √ | Study population | Participants with ALPPS and TSH. |
| **Reporting of search strategy should include** | |  |
| √ | Qualifications of searchers (eg, librarians and investigators) | The credentials of the two investigators Z.Z. and M.X.X. are provided in the author list. |
| √ | Search strategy, including time period included in the synthesis and keywords | A search of Pubmed, Embase and Cochrance Library databases was performed on all studies comparing ALPPS and TSH from 2009 to 2016. The following terms were searched: “liver partition”, “liver transection”, “portal vein occlusion”, “portal vein embolization”，“PVE”, “portal vein ligation”, “PVL”, “associating liver partition and portal vein ligation for staged hepatectomy”, “ALPPS” and “staged hepatectomy”. The publication time was defined from January 1, 2009 to May 31, 2017. |
| √ | Effort to include all available studies, including contact with authors | References of all retrieved articles and recent reviews were reviewed. |
| √ | Databases and registries | PubMed, Embase and Cochrance Library database |
| √ | Search software used, name and version, including special features used (eg, explosion) | We did not employ a special search software. |
| √ | Use of hand searching (eg, reference lists of obtained articles) | References of all retrieved articles and recent reviews were reviewed. |
| √ | List of citations located and those excluded, including justification | Details of the literature search process are outlined in the flow chart. |
| √ | Method of addressing articles published in languages other than English | We placed restrictions on English. |
| √ | Method of handling abstracts and unpublished studies | The search process was not restricted upon full-text articles, but also conference abstracts and unpublished studies. |
| √ | Description of any contact with authors. | We contacted the authors of the included studies to ask them for additional information and unpublished data as needed. |
| **Reporting of methods should include** | |  |
| √ | Description of relevance or appropriateness of studies assembled for assessing the hypothesis to be tested | The inclusion criteria are presented in the “Search strategy and selection criteria” section. |
| √ | Rationale for the selection and coding of data (eg, sound clinical principles or convenience) | We extracted the characteristics of each included study, including author, study design, sample size, Tumor size, Comparable variables,Quality score. |
| √ | Documentation of how data were classified and coded (eg, multiple raters, blinding, and inrerrater reliability) | Data were independently extracted and analyzed by two investigators (Z.Z. and M.X.X.) and final decision was reached by consensus. |
| √ | Assessment of study quality, including blinding of quality assessors; stratification or regression on possible predictiors of study results | The quality of each study was assessed by two investigators (Z.Z. and M.X.X.), using the Newcastle-Ottawa Scale. |
| √ | Assessment of heterogeneity | The *Q*-statistic and *I*-squared (*I*^2^) statistic were used to explore the heterogeneity among studies. |
| √ | Description of statistical methods (eg, complete description of fixed or random effects models, justification of whether the chosen models | Description of methods of meta-analyses and assessment of publication bias are detailed in the “Statistical analysis” section. |
| √ | Provision of appropriate tables and graphics | One main table is provided. One flow chart and 14 forest plots appear in the main text. |
| **Reporting of results should include** | |  |
| √ | Graph summarizing individual study estimates and overall estimate | Figure 1 |
| √ | Table giving descriptive information for each study included | Table 1 |
| √ | Indication of statistical uncertainty of findings | 95% confidence intervals were presented with all summary effect estimates. |
| **Reporting of discussion should include** | |  |
| √ | Quantitative assessment of bias (eg, publication bias) | “Results” section and “Discussion” section. |
| √ | Justification for exclusion (eg, exclusion of non-English-language citations) | The details of the exclusion of studies are shown in Flow chart. |
| √ | Assessment of quality of included studies | Table S2 |
| **Reporting of conclusions should include** | |  |
| √ | Consideration of alternative explanations for observed results | We discussed that the possibility of selection bias, misclassification bias related to exposure, and failure to consider residual or unmeasured confounding cannot be ruled out, thus, we cannot exclude chance, residual or unmeasured confounding as alternative explanation for our findings. |
| √ | Generalization of the conclusions (ie, appropriate for the data presented and within the domain of the literature review) | We discussed that the true effect would vary between the studies because of potential additional heterogeneity. Therefore, our results must be interpreted with caution. |
| √ | Guidelines for future research | We discussed that the investigators in the future study should improve the standardization of ALPPS. However, the safety and patients outcomes still need attention, meanwhile patient-selection was also of great importance in ALPPS. |
| √ | Disclosure of funding source | The authors didn’t receive any funding for this work. |
