# Supplementary material for: Associating liver partition and portal vein ligation for staged hepatectomy versus conventional two-stage hepatectomy: a systematic review and meta-analysis
Source: World J Surg Oncol. 2017 Dec 19;15:227. doi: 10.1186/s12957-017-1295-0 (PMC5738171; doi:10.1186/s12957-017-1295-0)
Supplement: Supplementary file 3 — Assessment of quality. (DOCX 15 kb) [file 12957_2017_1295_MOESM3_ESM.docx]

| Study | Selection | | | | Comparability | | Outcome | | | Quality score |
| --- | --- | --- | --- | --- | --- | --- | --- | --- | --- | --- |
|  | Representative treatment group | Representative reference group | Exposure determination | Outcome event before stare of research | Comparable for 1,2,3,4 | Comparable for 5,6,7,8,9 | Assignment of outcome | Adequate follow-up | Complete follow-up |  |
| Knoefel | Yes | Yes | Yes | No | 1,3 | 7 | Yes | No | No | 6 |
| Shindoh | Yes | Yes | Yes | No | 1,2 | 5,7 | Yes | Yes | No | 7 |
| Croome | Yes | Yes | Yes | No | 1,2 | 5,7,9 | Yes | No | Yes | 8 |
| Schadde | Yes | Yes | Yes | No | 1,2,4 | 5,7,8,9 | Yes | Yes | Yes | 9 |
| Ratti | Yes | Yes | Yes | No | 1,2 | 5,6,7,8,9 | Yes | Yes | Yes | 9 |
| Matsuo | Yes | Yes | Yes | No | 1,2,3 | 5,7 | Yes | No | No | 6 |
| Tanaka | Yes | Yes | Yes | No | 1,2 | 7 | Yes | No | Yes | 7 |
| Adam | Yes | Yes | Yes | Yes | 1,2 | 5,6,7,8 | Yes | Yes | Yes | 8 |
| Chia | Yes | Yes | Yes | No | 1,2,4 | 5,7 | Yes | Yes | No | 7 |
| Sandstrom | Yes | Yes | Yes | No | 1,2,3 | 5,7,8,9 | Yes | Yes | Yes | 8 |

1.Age 2.Preoperative chemotherapy 3.Preoperative liver function 4.Pathological state of liver before operative 5.pathological type of tumor 6.Pathology staging 7.sex 8. ASA score 9.BMI
